# Piperacillin-tazobactam induced immune hemolytic anemia led to increased renal impairment and eventual death from multiple organ failure in a patient with hypertensive nephropathy: case report and literature review

**DOI:** 10.1186/s12882-023-03235-w

**Published:** 2023-06-14

**Authors:** Yong Wu, Yuanjun Wu, Ganping Guo, Jiajun Zeng, Yan Liu, Yueqin Wu

**Affiliations:** 1 Department of Blood Transfusion, Dongguan Tungwah Hospital, Dongguan, China; 2 Department of Blood Transfusion, Dongguan Maternal and Child Health Hospital, Dongguan, China; 3 Department of Laboratory Medicine, Dongguan Maternal and Child Health Hospital, Dongguan Key Clinical Specialist, Dongguan, China

**Keywords:** Piperacillin, Drug-dependent antibody, Antiglobulin test, Drug-induced immune hemolytic anemia (DIIHA), Renal dysfunction after administration

## Abstract

**Background:**

Piperacillin is one of the most common drugs that cause drug-induced immune hemolytic anemia, but a complete description of the serological features and course of the disease is rare. This study completely describes the serological characteristics and course of a patient with hypertensive nephropathy who developed drug-induced immune hemolytic anemia and worsened renal function during repeated administration of piperacillin-tazobactam.

**Case presentation:**

A 79-year-old male patient with hypertensive nephropathy who developed severe hemolytic anemia and worsened renal function during intravenous piperacillin-tazobactam anti-infective treatment due to lung infection. Serological tests showed that the result of the direct antiglobulin test for anti-IgG was positive (4 +) and anti-C3d was negative, and the irregular red blood cell antibody screening test was negative. Plasma samples collected at different times from 2 days before to 12 days after the discontinuation of piperacillin-tazobactam administration were incubated with piperacillin solution and red blood cells of O-type healthy blood donors at 37 °C, IgG piperacillin-dependent antibodies were detected, and the highest titer was 128. However, no tazobactam-dependent antibody was detected in any plasma samples. Therefore, the patient was diagnosed with piperacillin-induced immune hemolytic anemia. Although blood transfusion and continuous renal replacement therapy were given, the patient died of multiple organ failure 15 days after the administration of piperacillin-tazobactam was stopped.

**Conclusion:**

This is the first complete description of the disease course and serological changes of piperacillin-induced immune hemolytic anemia, which is bound to help deepen the understanding of drug-induced immune hemolytic anemia and draw profound lessons from it.

## Background

Drug-induced immune hemolytic anemia (DIIHA) is a side effect of medication that can cause serious consequences. The annual incidence of DIIHA with obvious anemia is 1–4 per million people [1–3]. The mechanism of DIIHA is due to the immune damage to red blood cells (RBCs) caused by drug-induced antibodies (including drug-dependent antibodies and/or drug-independent antibodies) or non-immunologic protein adsorption (NIPA) [4–8]. It has been reported that nearly 140 kinds of drugs can cause DIIHA through drug-induced antibodies, and 10 kinds of drugs with NIPA effect [5–22].

Piperacillin is semi-synthetic penicillin against pseudomonas, which belongs to the β-lactam broad-spectrum antibiotics and exerts bactericidal activity by inhibiting the synthesis of sensitive bacterial cell walls. Some pathogenic bacteria can produce β-lactamase, which makes them resistant to β-lactam antibiotics. Tazobactam is an irreversible competitive β-lactamase inhibitor. The combined administration of piperacillin and tazobactam can prevent the pathogens producing β-lactamase from being resistant to piperacillin, thereby expanding the antibacterial spectrum of piperacillin and improving the antibacterial effect. Piperacillin-tazobactam has been widely used for bacterial infections [23]. However, piperacillin is one of the most common drugs that cause DIIHA and can cause fatal hemolytic anemia [3, 24, 25]. Tazobactam has been confirmed to have NIPA effects and can cause mild hemolysis [6, 7]. Here we report a 79-year-old male patient with hypertensive nephropathy who developed severe hemolytic anemia during intravenous piperacillin-tazobactam anti-infective treatment due to lung infection. The serological test detected piperacillin-dependent antibodies and the patient was diagnosed with piperacillin-induced DIIHA. Blood transfusion and continuous renal replacement therapy (CRRT) were given. However, 15 days after stopping the administration of piperacillin-tazobactam, the patient died of multiple organ failure. To deepen the understanding of DIIHA and to draw lessons from the case, we fully describe the patient's course of the disease and the serological changes after stopping the administration of piperacillin-tazobactam and reviewed the relevant literature.

## Case presentation

### Medical history of the patient

**Fig. 1:**
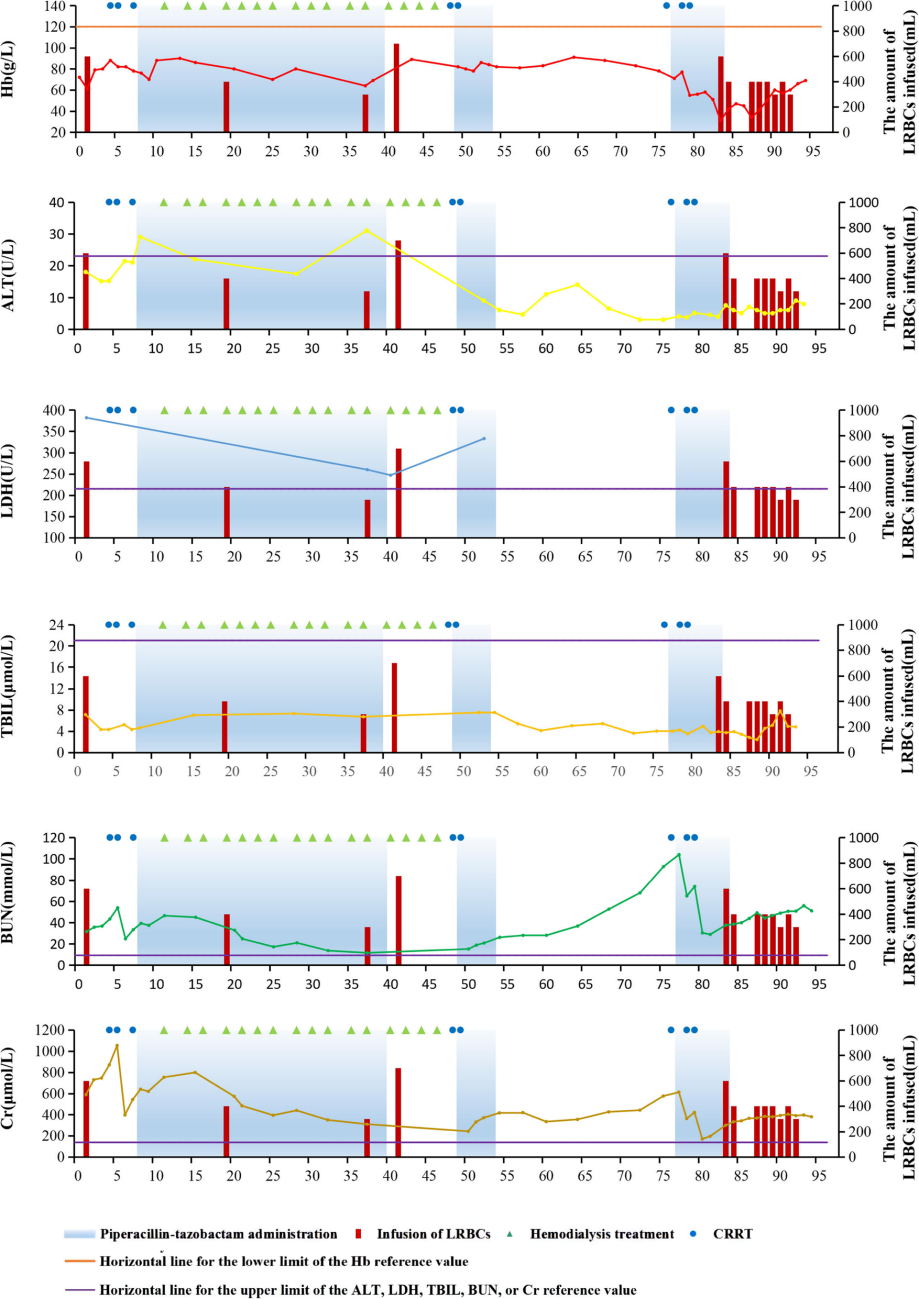
The dynamics of hemoglobin (Hb), alanine transaminase (ALT), lactate dehydrogenase (LDH), total bilirubin (TBIL), blood urea nitrogen (BUN), creatinine (Cr), and piperacillin-tazobactam administration. The amount of LRBCs infused: The amount of whole blood needed to prepare leukocyte-reduced red blood cells which were infused. Since the patient’s clinician in charge did not monitor the patient’s lactate dehydrogenase after the second course of piperacillin-tazobactam administration, the lactate dehydrogenase data is incomplete. LRBCs: leukocyte-reduced red blood cells. CRRT: continuous renal replacement therapy

A 79-year-old man was admitted to the hospital with "hypertension for more than 20 years, hypertensive nephropathy for 4 years, vomiting for 9 h, and urinary incontinence for 5 h". The clinical characteristics of the patient were as follows: hemoglobin (Hb) 72 g/L (reference values 120–160 g/L); alanine transaminase (ALT) 18U/L (reference values < 23 U/L); total bilirubin (TBIL) 7.1 μmol/L (reference values 2-21 μmol/L); lactate dehydrogenase (LDH) 382 U/L (reference values 135–215 U/L); blood urea nitrogen (BUN) 31.47 mmol/L (reference values 3.0–9.2 mmol/L); creatinine (Cr) 588.3 μmol/L (reference values 71–115 μmol/L); glucose-6-phosphate dehydrogenase (G6PD) activity, physiological levels. Through computer tomography (CT), the diagnosis was: left frontotemporal occipital subdural hematoma, left insular hemorrhage, acute exacerbation of the chronic obstructive pulmonary disease, pulmonary infection, hypertension grade 3 high-risk group, hypertension Nephropathy, chronic kidney disease stage 4, pericardial effusion. After admission, infused leukocyte-reduced red blood cells (LRBCs) prepared from 600 ml of whole blood, blood pressure control, hemostasis, erythropoietin, and other supportive treatments, hemodialysis once every 2–3 days, until the 50th day after admission Cr dropped to 242.6 μmol/L to end hemodialysis. On the 8th day after admission, the first course of piperacillin-tazobactam anti-infective treatment was given. Piperacillin-tazobactam 4.5 g was instilled intravenously every 12 h for 31 consecutive days for a total of 279 g of piperacillin-tazobactam. During and after the course of treatment, LRBCs prepared from 1400 ml of whole blood were infused. From 49 to 53 days after admission, he was given a second course of piperacillin-tazobactam anti-infective treatment. A total of 26 g piperacillin-tazobactam was injected, and Cr gradually increased to 575.5 μmol/L. On the 77th day after admission, the third course of piperacillin-tazobactam anti-infective treatment was started. During the period, Cr increased to 611.8 μmol/L and a total of 26 h CRRT was given twice. On the 7th day of the third course of treatment with piperacillin-tazobactam, Hb dropped to a minimum of 31 g/L. Because it was suspected to be related to the treatment of piperacillin-tazobactam, the administration of piperacillin-tazobactam was stopped on the 83rd day after admission. In the third course of treatment, a total of 63 g of piperacillin-tazobactam was injected. During the third course of treatment with piperacillin-tazobactam and within 10 days after the course of treatment, LRBCs prepared from 3200 ml of whole blood and virus-inactivated fresh frozen plasma 1050 ml were infused. The patient had no bleeding during hospitalization, and Hb was 66 g/L after the last blood transfusion. The patient died of multiple organ failure on the 97th day of admission (15 days after the administration of piperacillin-tazobactam was stopped). The dynamics of Hb, ALT, LDH, TBIL, BUN, Cr, and piperacillin-tazobactam administration are shown in Fig. 1.

### Serological test results

The results of the direct antiglobulin test (DAT) for anti-IgG with a Coombs card (Diagnostic Grifols, S.A.) of blood samples collected on days 2, 1, and 0 before stopping piperacillin-tazobactam administration were strongly positive (4 +). After stopping the administration of piperacillin-tazobactam, the DAT for anti-IgG results gradually weakened and turned negative until 12 days after the administration of piperacillin-tazobactam was stopped. The results of the DAT for anti-C3d (Shanghai Blood Biomedical Co., Ltd., Shanghai, China) with the tube method of all blood samples were negative. The irregular RBC antibody test using plasma was weakly positive before stopping and became negative after stopping piperacillin-tazobactam administration. The weakly positive results may be due to the presence of piperacillin in the patient's plasma and the piperacillin-dependent antibodies have agglutinated with the RBCs. The irregular RBC antibody tests using the acid eluent (acid elution reagents were produced by Guangzhou Zhanquan Biotech Co., Ltd) were collected before and after the patient stopped the administration of piperacillin-tazobactam were all negative. The detailed results of DAT and irregular RBC antibody screening are shown in Table 1. Following the previous reports [26, 27], detection of drug-dependent antibodies in the presence of a drug solution and using drug-coated RBCs were performed. In a previous study [27], piperacillin may be more suitable for coating RBCs under high pH and room temperature conditions. According to the materials available in the laboratory, we used phosphate buffer solution (PBS) with pH 7.2 and 9.0 to prepare piperacillin solutions and used PBS with pH 7.2 to prepare tazobactam solution, and the concentration of the drug solutions was all 40 mg/ml. These drug solutions was each incubated with O-type donor washed red blood cells (WRBCs, DAT result was negative and without drug treatment) at room temperature and 37 °C for 1 h individually, to prepare drug-coated RBCs. Plasma samples of the patient collected on day 2 before and day 3 after stopping piperacillin-tazobactam administration was each incubated with O-type donor WRBCs and piperacillin solution (3 mg/mL) at 37℃ for 1 h, observed after centrifugation, and there were no hemolysis or agglutination, but the anti-globulin tests performed with a monospecific anti-IgG Coombs card were both positive (4 +). A plasma sample of the patient collected on day 2 before stopping piperacillin-tazobactam administration was incubated with O-type donor WRBCs, or piperacillin-coated RBCs, or tazobactam-coated RBCs at 37℃ for 1 h, observed after centrifugation, and there were no hemolysis or agglutination, but the anti-globulin tests performed with a monospecific anti-IgG Coombs card were all positive (3 +). However, plasma sample of the patient collected on day 3 after stopping piperacillin-tazobactam administration was incubated with O-type donor WRBCs, or piperacillin-coated RBCs, or tazobactam-coated RBCs at 37℃ for 1 h, observed after centrifugation, and there were no hemolysis or agglutination, and the anti-globulin tests performed with a monospecific anti-IgG Coombs card were all negative. Therefore, it is judged that there were IgG piperacillin-dependent antibodies that can only be detected in piperacillin solution and do not react with piperacillin-coated RBCs in the patient's plasma. Plasma sample collected on day 2 before stopping piperacillin-tazobactam administration was incubated with O-type donor WRBCs, or piperacillin-coated RBCs, or tazobactam-coated RBCs at 37℃ for 1 h, the results of the anti-globulin tests were positive because of the presence of piperacillin in the patient's plasma. No tazobactam-dependent antibody was detected in the plasma samples and acid eluent collected before and after the patient stopped the administration of piperacillin-tazobactam, and no piperacillin-dependent antibody was detected in the acid eluent of the patient. The detailed results of drug-dependent antibody detection in patient's plasma and acid eluent are shown in Table 2. The titers of IgG piperacillin-dependent antibodies in plasma samples collected 2 days before to 12 days after stopping piperacillin-tazobactam administration ranged from 64 to 128, as shown in Table 1.

**Table 1:** Results of DAT, irregular RBC antibody screening, and the titers of piperacillin-dependent antibody

After stopping the administration of piperacillin-tazobactam (day)	Direct antiglobulin test (anti-IgG)^a^	Irregular RBC antibody	Titers of piperacillin-dependent antibodies
-2	4 +	weak positive^b^	64
-1	4 +	weak positive^b^	not tested
0	4 +	weak positive^b^	not tested
1	3 +	negative	not tested
2	3 +	negative	64
3	2 +	negative	not tested
5	2 +	negative	not tested
6	1 +	negative	128
9	1 +	negative	128
10	weak positive	negative	64
11	weak positive	negative	64
12	negative	negative	64

^a^The blood samples collected at each time point were subjected to the direct antiglobulin test for anti-C3d, and the test results were all negative

^b^The weak positive results of irregular RBC antibody screening tests were due to the presence of piperacillin in the patient's plasma, and piperacillin-dependent antibodies lead to agglutination of red blood cells of the antibody screening reagent. + : strong

**Table 2:** Test results of drug-dependent antibodies in blood samples collected 2 days before and 3 days after stopping the administration of piperacillin-tazobactam

NO.	Reactive materials								Results of the antiglobulin test^a^	
	P-P (μl)	AE (μl)	AB-P (μl)	3 mg/ml PRC (μl)	1 mg/ml TBT (μl)	RBCs (μl)	PRC-RBCs (μl)	TBT-RBCs (μl)	2 days before stopping	3 days after stopping
1	100	/	/	100	/	50	/	/	4 +	4 +
2	100	/	/	/	100	50	/	/	-	-
3	/	100	/	100	/	50	/	/	-	-
4	/	100	/	/	100	50	/	/	-	-
5	100	/	/	/	/	50	/	/	3 + ^b^	-
6	100	/	/	/	/	/	50	/	3 + ^b^	-
7	100	/	/	/	/	/	/	50	3 + ^b^	-
8	/	100	/	/	/	50	/	/	-	-
9	/	100	/	/	/	/	50	/	-	-
10	/	100	/	/	/	/	/	50	-	-
11	/	/	100	100	/	50	/	/	-	-
12	/	/	100	/	/	50	/	/	-	-
13	/	/	100	/	/	/	50	/	-	-
14	/	/	100	/	/	/	/	50	-	-

*P-P* Patient’s plasma, *AE* Patient’s acid eluent, *AB-P* AB-type plasma with negative antibody screening test result, *PRC* Piperacillin, *TBT* Tazobactam, *RBCs* Uncoated O-type red blood cells, *PRC-RBCs* Piperacillin-coated red blood cells, *TBT-RBCs* Tazobactam-coated red blood cells, *2 days before stopping* 2 days before stopping the administration of piperacillin-tazobactam, *3 days after stopping* 3 days after stopping the administration of piperacillin-tazobactam

^a^The same methods were used to detect drug-dependent antibodies in blood samples collected on day 2 before and on day 3 after the patient’s stopping of the administration of piperacillin-tazobactam, and the direct antiglobulin tests were used to confirm the presence of drug-dependent antibodies

^b^Due to the presence of piperacillin in the patient’s plasma before stopping the administration of piperacillin-tazobactam, the plasma reacted with both drug-coated red blood cells and uncoated red blood cells; + : strong; -: negative

Remarks: All the 14 combinations of reactive materials were incubated at 37ºC for 1 h and observed by centrifugation (no agglutination and hemolysis were found), and then performed the antiglobulin tests to detect drug-dependent antibodies

## Discussion and conclusion

Diagnosis of DIIHA includes the positive results of DAT for anti-IgG and/or for anti-C3d, detection of drug-induced antibodies, including drug-dependent antibodies, and/or drug-independent antibodies which non-existent before administration, but induced by the drug after administration, and it can be traced back to the time-dependent immune hemolysis [5, 26]. In this patient, the serological test results showed that DAT for anti-IgG was strongly positive (4 +). There were IgG piperacillin-dependent antibodies in the plasma detected after incubation with piperacillin solution and RBCs at 37 °C. However, no irregular RBC antibody and tazobactam-dependent antibody were detected in the patient's plasma, and no antibody was detected in the acid eluent. The clinical manifestations were hemolytic anemia and aggravation of renal dysfunction with a clear time correlation with piperacillin-tazobactam administration. Therefore, the patient can be diagnosed with DIIHA caused by piperacillin, and DIIHA was responsible for his increased renal impairment.

**Fig. 2:**
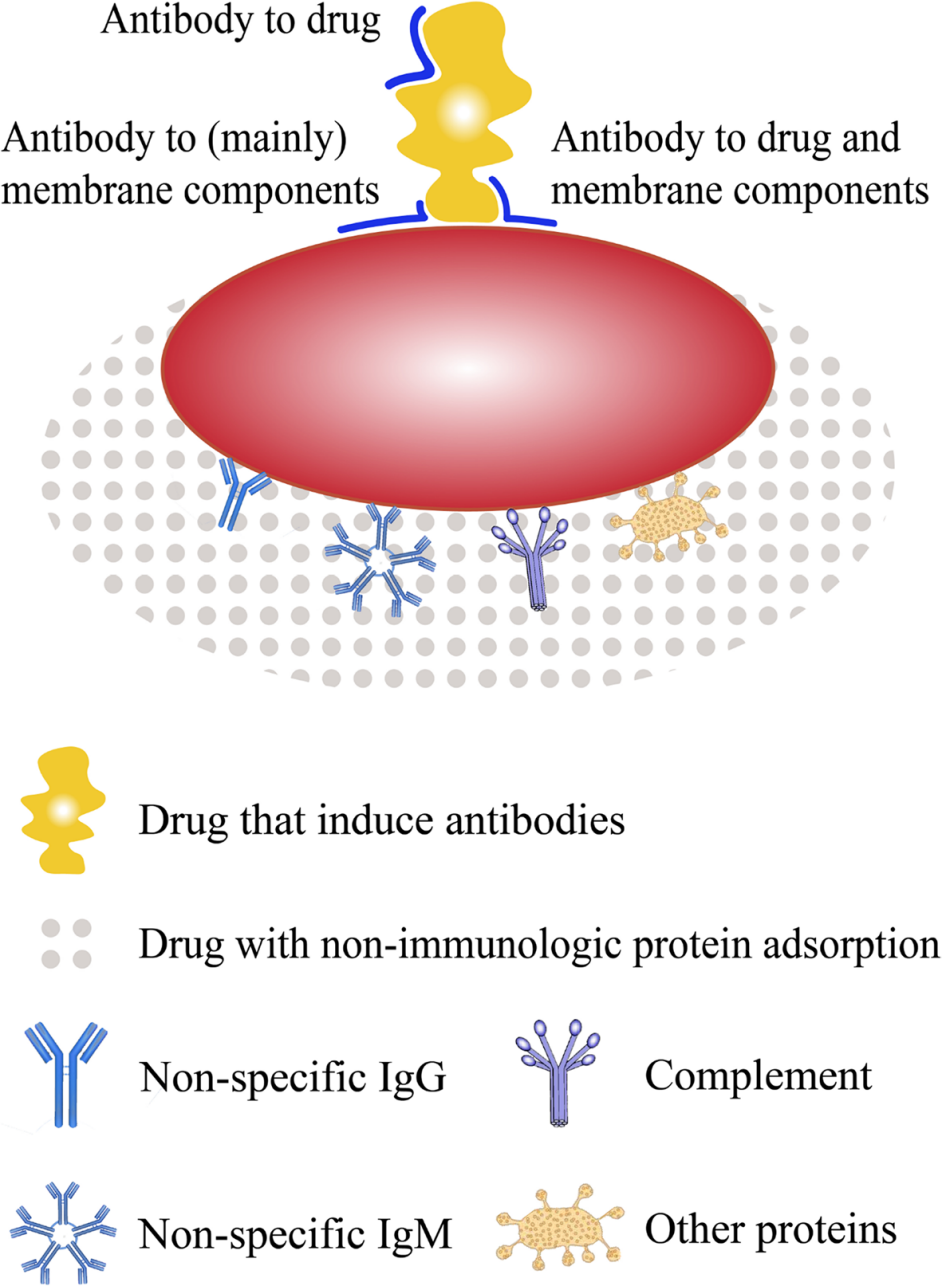
Current understanding of the mechanism of drug-induced immune hemolytic anemia (DIIHA). The mechanisms of DIIHA include drug-induced antibodies and non-immunologic protein adsorption (NIPA). Drug-induced antibodies include the following 4 reaction modes: (1) Drug only, this is the characteristic of the drug adsorption mechanism, and may be suitable for detection with drug-coated red blood cells; (2) Drug + membrane, this is a typical immune complex mechanism, may be suitable for detection in the presence of a drug solution; (3) Mainly membrane, this is the drug-independent antibody similar to IgG warm auto-antibodies and can be detected by antibody screening test in the absence of drug; (4) A combination of the first three, reactive under a variety of test conditions. Drug-induced antibodies are the main mechanism of severe DIIHA. NIPA is a drug that modified the red blood cell membrane so that plasma proteins (including immunoglobulins such as IgM and IgG, complement, albumin, etc.) were adsorbed nonimmunologically, can only cause a positive result of the direct antiglobulin test and slow, difficult to observe slight hemolysis

Before the 1980s, Garratty et al. described DIIHA as the following four mechanisms [5, 28]: (1) Immune Complex, which is drug and drug antibodies combined to form immune complexes, which were then nonspecifically adsorbed onto RBCs and activated complement, the representative drug is quinidine; (2) Drug Adsorption, which is antibodies to the drug that reacted with the RBC-bound drug, the representative drug is penicillin; (3) NIPA, which is a drug that modified the RBC membrane so that plasma proteins were adsorbed nonimmunologically, the representative drugs are β-Lactamase inhibitors; (4) Autoimmune hemolytic anemia (AIHA), which is a drug-induced autoantibody that reacted with normal RBCs (no drug added), similar to reactivity seen with auto-antibodies found in idiopathic IgG warm AIHA, the representative drug is methyldopa. With the gradual understanding of the mechanism of DIIHA, it is now believed that the mechanisms of DIIHA include drug-induced antibodies and NIPA. Drug-induced antibodies include the following 4 reaction modes [5, 29, 30]: (1) Drug only, this is the characteristic of the drug adsorption mechanism, the representative drug is penicillin; (2) Drug + membrane, this is a typical immune complex mechanism, the representative drug is quinidine; (3) Mainly membrane; (4) A combination of the first three. Figure 2 illustrates the current understanding of the mechanism of DIIHA. Drugs that are loosely or firmly bound to cell membranes form immune complexes with drug-induced antibodies, cells bound with drug-antibody immune complexes are phagocytosed by macrophages, and/or complement is activated to cause cytolysis [5]. Almost all DIIHA that is manifested as significant anemia is caused by drug-induced antibodies [4, 5]. The NIPA mechanism can only cause a positive result of the DAT and slow, difficult to observe slight hemolysis [6–8].

Drug-induced antibodies can also be divided into drug-dependent antibodies (reactivity is "drug only" and/or "drug + membrane") and drug-independent antibodies (reactivity is "mainly membrane"). Drug-dependent antibodies must react in vivo or in vitro only in the presence of related drug or its metabolite. Drug-independent antibodies are similar to warm autoantibodies against RBCs and can react in vivo or in vitro in the absence of related drug that induce antibodies and its metabolite [5, 26]. The majority of DIIHA with obvious hemolysis is caused by drug-dependent antibodies, which are manifested as immune hemolytic anemia with a clear time correlation with the relevant administration. After the administration is stopped, the hemolysis is relieved and gradually stopped. It is very difficult to distinguish between drug-independent antibodies and warm autoantibodies against RBCs and to diagnose DIIHA caused by drug-independent antibodies. The duration of immune hemolytic anemia caused by drug-independent antibodies after stopping the administration of related drugs is not clear [31, 32]. We observed one case of DIIHA induced by oral cimetidine with immune hemolytic anemia that persisted until 41 days after the cimetidine administration was stopped due to the presence of drug-independent non-specific antibodies [33].

Piperacillin is the most immunogenic drug [34, 35] and the most common antibacterial drug reported to cause DIIHA. There have been dozens of cases of severe DIIHA caused by piperacillin, including deaths. These cases are all caused by the immune-complex mechanism, that is, the antibodies induced by piperacillin [3, 24, 25, 36–41]. In the reported cases of DIIHA caused by piperacillin diagnosed by standard serological tests, almost all of them have detected positive results of DAT for anti-IgG and no blood group antigen-specific piperacillin-dependent antibodies [3, 24, 25, 36–43]. Most of these cases show acute intravascular hemolysis because piperacillin-dependent antibodies can activate complement [3, 24, 36–41].

There are many reasons for the positive DAT result [31], but in DIIHA, the positive result of DAT for anti-C3d may reflect that the drug-induced antibodies can activate complement and cause acute intravascular hemolysis. Among the 8 cases of severe DIIHA caused by piperacillin reported by Mayer et al. [36], the lowest value of Hb was 7.7 g/dL in one case with negative DAT for anti-C3d result, and the lowest Hb values of the other 7 patients with positive DAT for anti-C3d results were all lower than 7.7 g/dL, it suggests that DAT for anti-C3d may be related to the severity of DIIHA. However, in this case, although the results of continuous monitoring of DAT for anti-C3d were negative after the diagnosis of DIIHA was confirmed, the patient’s hemolysis was severe (the lowest value of Hb detected was 31 g/L), which suggests that severe DIIHA can occur in patients with the negative result of DAT for anti-C3d.

In the reported cases of DIIHA caused by piperacillin, drug-independent antibodies induced by piperacillin are rarely detected. In a prospective study of the risk of DIIHA caused by piperacillin in patients with cystic fibrosis and anti-pseudomonas infection treated with piperacillin, one patient was receiving a piperacillin-tazobactam combination, during the treatment, a mild immune hemolytic anemia occurred, and this patient was detected Rhe-specific autoantibodies. But after dialysis to remove piperacillin in the sample, the antibodies could not be detected, and with the addition of piperacillin or its metabolite (urine during piperacillin treatment), the autoantibodies with Rhe specificity can be detected. This study shows that the antibodies are also piperacillin-dependent antibodies [3].

Methods to detect drug-dependent antibodies include detection with drug-coated RBCs and detection in the presence of soluble drugs [5, 26]. Since the mechanism of drug-dependent antibodies production may be different, different drug-dependent antibodies may be suitable for detection by different methods [9]. The mechanism of DIIHA caused by penicillin is due to the immune damage of penicillin-dependent antibodies produced by the body to RBCs coated with penicillin. Patients with DIIHA caused by penicillin are suitable for penicillin-coated RBCs to detect penicillin-dependent antibodies [26, 27]. Although piperacillin is semi-synthetic penicillin, piperacillin-dependent antibodies have different serological characteristics from penicillin-dependent antibodies. A serological study on piperacillin-dependent antibodies [27] showed the plasma of 100 blood donors was each incubated with the piperacillin-coated RBCs at 37 °C for 1 h, and IgM piperacillin-dependent antibodies were detected in the plasma of 91 blood donors. And these antibodies can be completely inhibited by 10 mg/ml of piperacillin. The detection of IgM piperacillin-dependent antibodies in the plasma of healthy blood donors was interpreted as a result of immunization with piperacillin or related chemicals exposed to the environment. However, 6 patients with DIIHA caused by piperacillin observed during the same period could only detect piperacillin-dependent antibodies by incubating the patient’s plasma, piperacillin solution, and RBCs. But the piperacillin-dependent antibodies could not be detected by the piperacillin-coated RBCs. It is suggested that the piperacillin-dependent antibody produced by exposure to piperacillin or related chemicals in the environment and the piperacillin-dependent antibody induced by piperacillin administration have different serological characteristics. The reactivity of antibodies produced by immunization with piperacillin or related chemicals exposed to the environment is "drug only", while the reactivity of antibodies produced by immunization with piperacillin administration is "drug + membrane". The clinical significance of piperacillin-dependent antibodies produced by exposure to piperacillin or related chemicals in the environment is unclear.

According to the methods of the previous reports [26], 1 mg/ml is the standard drug concentration used to detect drug-dependent antibodies in the presence of the soluble drugs. However, a serological study by Leger et al. [27] on piperacillin-dependent antibodies showed that piperacillin solutions with a concentration of 1–10 mg/ml can detect piperacillin-dependent antibodies in patients with DIIHA caused by piperacillin, but when there is a high concentration of piperacillin, piperacillin-dependent antibody response will be stronger. Therefore, we used 3 mg/ml piperacillin solution to detect piperacillin-dependent antibodies, and 1 mg/ml tazobactam solution to detect the presence of tazobactam-related antibodies.

The patient in this study, Hb72g/L before administration of piperacillin-tazobactam, three courses within 3 months, total intravenous administration of piperacillin-tazobactam 368 g, during which a total of LRBCs prepared from 5200 ml of whole blood were infused, and Hb 66 g/L after the last infusion of LRBCs. The patient had severe hypertensive renal impairment. During the first course of piperacillin-tazobactam administration, because multiple times CRRT, hemodialysis, blood transfusion, and other treatments were given, and the strength of the piperacillin-dependent antibodies may be low, he did not show obvious clinical characteristics of DIIHA. Significantly aggravated renal damage occurred after the CRRT was stopped during the second and the third courses of piperacillin-tazobactam administration, while severe hemolytic anemia occurred in the third course of piperacillin-tazobactam and within a few days after the end of the third course, possibly because the patient repeated immunization with piperacillin increased the strength of piperacillin-dependent antibodies. Due to insufficient knowledge of DIIHA and lack of necessary vigilance [3, 10], when the patient experienced aggravated renal damage and severe hemolytic anemia, the administration of piperacillin-tazobactam could not be stopped in time, which led to the patient's condition deteriorated rapidly after the third course of piperacillin-tazobactam administration. Although the diagnosis of DIIHA due to piperacillin was confirmed by serological testing after consultation with an immunohematologist, and the administration of piperacillin-tazobactam was discontinued. However, the patient eventually died of multiple organ failure. Predisposing factors to multiple organ failure may include infection, but severe DIIHA should be a more important factor. Based on the lessons of this case and the recommendations previously reported, DIIHA caused by piperacillin has been regarded as a highly acute and life-threatening event, particularly if it remains undetected in due time [2]. Any patient who develops hemolytic anemia during the administration of piperacillin should consider the possibility of DIIHA. A timely serological test to confirm the diagnosis and stop the administration of piperacillin is the most effective strategy to avoid the serious consequences of DIIHA caused by piperacillin.

We recently reported a case of severe acute intravascular hemolysis in a neonate with ABO-incompatible hemolytic disease of the newborn following administration of cefotaxime-sulbactam. It was confirmed that the NIPA effect of sulbactam promoted the specific binding of maternally derived incompatible ABO blood group antibodies with the neonatal RBC blood group antigen, thereby activating complement [11]. Both tazobactam and sulbactam are β-Lactamase inhibitors with NIPA effect. Whether the NIPA effect can promote the reactivity of drug-induced antibodies is unclear.

As far as we know, this is the first case of DIIHA caused by piperacillin with complete tracking of serological changes and the disease processes, but lack of reticulocyte count and haptoglobin data, and incomplete LDH monitoring. Delays in diagnosis due to insufficient understanding of DIIHA are worth learning a lesson. DIIHA is a side effect of medication that can lead to serious consequences, even fatal. Due to the low incidence of severe DIIHA, it has not yet received the attention of clinicians, clinical pharmacists, and drug manufacturers. In China, DIIHA has not yet been included in the medication risk monitoring system. It is now clear that there are nearly 140 drugs that can cause DIIHA through drug-induced antibodies [5, 9–22, 33], but DIIHA has not been included in the list of side effects of these drugs. Due to a lack of vigilance against DIIHA, the vast majority of DIIHA may be missed, misdiagnosed, delayed in diagnosis, or given improper intervention, which increases the risk of patient administration. Incorporating DIIHA into the medication risk monitoring system and improving the awareness and vigilance of DIIHA will help avoid the serious consequences of DIIHA on patients.

## Data Availability

The datasets generated during and/or analysed during the current study are available from the corresponding author on reasonable request.

## Abbreviations

DIIHA: Drug-induced immune hemolytic anemia
RBC: Red blood cell
NIPA: Non-immunologic protein adsorption
CRRT: Continuous renal replacement therapy
Hb: Hemoglobin
ALT: Alanine transaminase
TBIL: Total bilirubin
LDH: Lactate dehydrogenase
BUN: Blood urea nitrogen
Cr: Creatinine
G6PD: Glucose-6-phosphate dehydrogenase
CT: Computer tomography
LRBCs: Leukocyte-reduced red blood cells
DAT: Direct antiglobulin test
WRBC: Washed red blood cell
PBS: Phosphate buffer solution
AIHA: Autoimmune hemolytic anemia
